# Bisdemethoxycurcumin Increases Sirt1 to Antagonize *t*-BHP-Induced Premature Senescence in WI38 Fibroblast Cells

**DOI:** 10.1155/2013/851714

**Published:** 2013-09-02

**Authors:** Ying-Bo Li, Zhang-Feng Zhong, Mei-Wan Chen, Jiao-Lin Bao, Guo-Sheng Wu, Qing-Wen Zhang, Simon Ming-Yuen Lee, Pui-Man Hoi, Yi-Tao Wang

**Affiliations:** ^1^State Key Laboratory of Quality Research in Chinese Medicine, Institute of Chinese Medical Sciences, University of Macau, Avenue Padre Tomás Pereira S.J., Taipa 999078, Macau; ^2^State Key Laboratory of Natural and Biomimetic Drugs, School of Pharmaceutical Sciences, Peking University, Beijing 100191, China

## Abstract

Curcuminoids are well known for their capabilities to combat risk factors that are associated with ageing and cellular senescence. Recent reports have demonstrated that curcuminoids can extend the lifespan of model organisms. However, the underlying mechanisms by which these polyphenic compounds exert these beneficial effects remain unknown. In this study, *t*-BHP-induced premature senescence model in human fibroblasts was chosen to explore the protective effects of a curcuminoid, bisdemethoxycurcumin (BDMC), on cellular senescence. The results demonstrated that BDMC attenuated oxidative stress-induced senescence-like features which include the induction of an enlarged cellular appearance, higher frequency of senescence-associated **β**-galactosidase staining activity, appearance of senescence-associated heterochromatic foci in nuclei, decrease in proliferation capability, and alteration in related molecules such as p16 and retinoblastoma protein. Notably, we found that BDMC treatment activated Sirt1/AMPK signaling pathway. Moreover, downregulating Sirt1 by the pharmacological inhibitor nicotianamine or small interfering RNA blocked BDMC-mediated protection against *t*-BHP-mediated decrease in proliferation. These results suggested that BDMC prevented *t*-BHP-induced cellular senescence, and BDMC-induced Sirt1 may be a mechanism mediating its beneficial effects.

## 1. Introduction

 Curcuminoids, including curcumin, demethoxycurcumin, and bisdemethoxycurcumin (BDMC), are the main active components that are derived from the rhizome of *Curcuma longa*, which is a commonly used spice in curry dishes. Extensive studies have shown that curcuminoids possess a wide spectrum of therapeutic benefits with low toxicity. These include protection against inflammation, carcinogens, and cardiovascular and neurodegenerative diseases. Although these pathologies have individual complex mechanisms, ageing can contribute to the etiology and risk factors related to these age-associated disorders [1–3]. It was demonstrated that curcuminoids have potent antioxidative capability, including scavenging free radicals and enhancing antioxidant defense. Since oxidative stress and oxidative stress-induced damage accumulation are important trigger factors of cellular senescence and ageing [4, 5], it is possible that curcuminoids may exert protective roles in the ageing process. Indeed, it has been recently reported that the administration of curcumin could extend the lifespan of two strains of *Drosophila melanogaster* [6]. In addition, it was demonstrated that feeding with tetrahydrocurcumin, a major metabolite of curcumin, can increase the average lifespan of male C57BL/6 mice [7]. These observations present curcuminoids as attractive candidates in prevention of ageing and ageing-related disorders. However, the molecular mechanisms by which curcuminoids exert their effects remain unknown. 

Various studies highlighted the promising ability of polyphenolic compounds to combat risk factors associated with ageing and even to prevent cellular senescence and ageing. It has been shown that resveratrol, a well-studied polyphenol in red wine, can protect against oxidative stress-induced apoptosis, promote proliferation and prevent senescence in endothelial cells [8–10]. In addition, dietary administrations of resveratrol, quercetin, or blueberry polyphenols increased lifespan and stress resistance in *Caenorhabditis elegans* [11–14]. Notably, apart from the inhibitory effect against oxidative insults, modulation on specific molecules such as Sirt1 has been implicated as a major contributing factor for these functions of resveratrol and other polyphenolic compounds. Sirt1, a conserved NAD^+^-dependent deacetylase, is a primary mediator of cellular metabolism and lifespan extension following caloric restriction [15]. Increase in Sir2 (homolog of Sirt1) activity prolonged the lifespan of yeast, worms, and flies [16, 17], suggesting that Sirt1 plays a crucial role in ageing and ageing-related diseases. Since polyphenolic compounds, such as curcuminoids, have beneficial effects on ageing-associated disorders, this raised the possibility that Sirt1 functions as a mediator for the effects of curcuminoids. Therefore, in this study, we explored the effects of BDMC on oxidative stress-induced premature senescence as well as its effects on Sirt1. 

## 2. Materials and Methods

### 2.1. Reagents

Bisdemethyoxycurcumin (BDMC) was separated from *Curcuma longa* (turmeric). Its structure (Figure 2(d)) was deduced on the basis of their physicochemical properties and spectral data, and the purity of the compound was >98%. Basal medium Eagle medium (BME), fetal calf serum (FCS), *t*-BHP solution 70% in H_2_O, and 5-bromo-4-chloro-3-indolyl-*β*-D-galactopyranoside were supplied by Sigma-Aldrich (USA). MTT [3-(4,5-dimethyl-2-thiazolyl)-2,5-diphenyl tetrazolium bromide] was purchased from USB (USA). Hoechst 33342 dye was purchased from Molecular Probe (USA). 4′-6-Diamidino-2-phenylindole (DAPI) was purchased from Millipore (USA). Antibodies against p21 (F-5), p16 (C-20), and Sirt1 were purchased from Santa Cruz (USA). Antibodies against phosphor-AMPK, AMPK, phospho-ERK1/2, phospho-Rb, Rb, and *β*-actin were purchased from Cell Signaling Technology (USA). Nicotinamide was purchased from Beyotime (China). 

### 2.2. Cell Culture

The WI-38 human diploid fibroblasts (HDFs) were purchased from ATCC (USA). These cells were maintained in 37°C and 5% CO_2_ in BME supplemented 10% (v/v) FCS. The cells have a finite lifetime of 50 (plus or minus 10) population doublings (PD) with a doubling time of 24 hours and are considered to be young earlier than PD30 and fully senescent at PD50 or later. 

### 2.3. Drug Treatment

To induce premature senescence, subconfluent HDFs at early passage were treated five times for 1 h with 30 *μ*M *t*-BHP freshly diluted in BME with 10% FCS. At the end of each stress, the HDFs were washed with PBS and cultivated in fresh BME medium with 10% FCS. In the BDMC-treated group, cells were exposed to BDMC at different concentrations for the indicated period before senescence induction. BDMC was dissolved in DMSO and its concentration did not exceed 0.2% of the total volume.

### 2.4. Senescence-Associated *β*-Galactosidase (SA *β*-Gal) Staining

SA *β*-galactosidase-positive cells were detected as described previously [18]. HDFs were fixed for 3–5 min at room temperature in 3% formaldehyde. After being washed with PBS, cells were incubated at 37°C without CO_2_ with fresh SA-*β*-Gal staining solution: 1 mg of 5-bromo-4-chloro-3-indolyl-*β*-D-galactopyranoside per mL (stock = 20 mg of dimethylformamide per mL)/40 mM citric acid/sodium phosphate, pH 6.0/5 mM potassium ferrocyamide/5 mM potassium ferricyanide/150 mM NaCl/2 mM MgCl_2_. Cells were examined after 10–16 h incubation. 

### 2.5. Senescence-Associated Heterochromatic Foci (SAHF) Analysis

SAHF was determined as described previously [19]. Cells were fixed with 4% para formaldehyde followed by permeabilization with 0.1% Triton X-100 for 10 min. Then, nucleic acid was stained by DAPI (5 *μ*g/mL) for 3 min. After three washes with PBS, stained cells were examined using a fluorescent microscope (Carl Zeiss, Axiovert 200, USA).

### 2.6. Determination of Cell Proliferation

After drug treatment, HDFs were seeded at 2 × 10^3^ cells/well in 96-well plates and allowed to grow in BME with 10% FCS for another 72 h. Cells were then incubated with 100 *μ*L BME medium containing 0.5 mg/mL MTT. After 4 h incubation at 37°C, cell supernatants were discarded, MTT crystals were dissolved in 100 *μ*L DMSO, and absorbance was measured at 570 nm using a Multilabel counter (Perkin Elmer, 1420 Multilabel Counter Victor3, Wellesley, USA). The absorbance reflects the cell number after proliferation for 72 h with different treatment procedures.

### 2.7. Western Blot Analysis

Protein was extracted using RIPA lysis buffer with 1% phenylmethanesulfonyl fluoride and 1% protease inhibitor. Lysates were centrifuged at 12,000 ×g for 20 min at 4°C, and the supernatant was collected. Total protein concentrations were determined using BCA Protein Assay kit. Supernatants containing 40 *μ*g of protein/lane were separated by SDS-PAGE. After electrophoresis, the separated proteins were electrically transferred onto polyvinylidene difluoride membranes. The membrane was probed with a primary antibody followed by a second antibody and visualized using an ECL advanced Western blotting detection kit. Photos of protein bands were taken by Molecular Imager ChemiDoc XRS (Biorad, USA). Densitometric measurements of band intensity in the Western blots were performed using Quantity One Software.

### 2.8. Reverse Transcription and Real-Time PCR

Total RNA was isolated using Trizol reagent (Invitrogen, USA). First-strand cDNA were generated from RNA samples by reverse transcription using oligo (dT), followed by real-time PCR by LightCycler 480 Real-time PCR instrument (Roche, USA). The flowering primers were used to amplify fragments of the human Sirt1: 5′-GACTTCAGGTCAAGGGATGGT-3′ (F) and 5′-CAGCGTGTCTATGTTCTGGGTAT-3′ (R). GAPDH: 5′-CATGAGAAGTATGACCAACAGCCT-3′ (F) and 5′-AGTCCTTCCACGATACCAAAGT-3′ (R).

### 2.9. Short Interfering Ribonucleic Acid Transfection

The short interfering ribonucleic acid (siRNA) duplexes used in this study were chemically synthesized by GenePharma Corporation (Shanghai, China). 5′-CGGGAAUCCAAAGGAUAAUTT-3′ and 5′-AUUAUCCUUUGGAUUCCCGTT-3′ or 5′-GUAUGACAACAGCCUCAAGTT-3′ and 5′-CUUGAGGCUGUUGUCAUACTT-3′ were used to repress Sirt1 or GADPH expression, respectively. Nonsilencing siRNA (5′-UUCUCCGAACGUGUCACGUTT-3′ and 5′-ACGUGACACGUUCGGAGAATT-3′) was used as a negative control. siRNA duplexes were transfected into WI38 HDFs with Lipofectamine 2000 transfection reagent (Invitrogen, USA) according to the manufacturer's instructions.

### 2.10. Statistical Analysis

All results were presented as mean ± SD of three independent experiments. Data were analyzed by Student's *t-*test after analysis of variance. *P* < 0.05 was considered to be statistically significant.

## 3. Results

### 3.1. *t*-BHP Treatment Induced Changes of Senescence-Associated Features in WI38 HDFs

**Figure 1:**
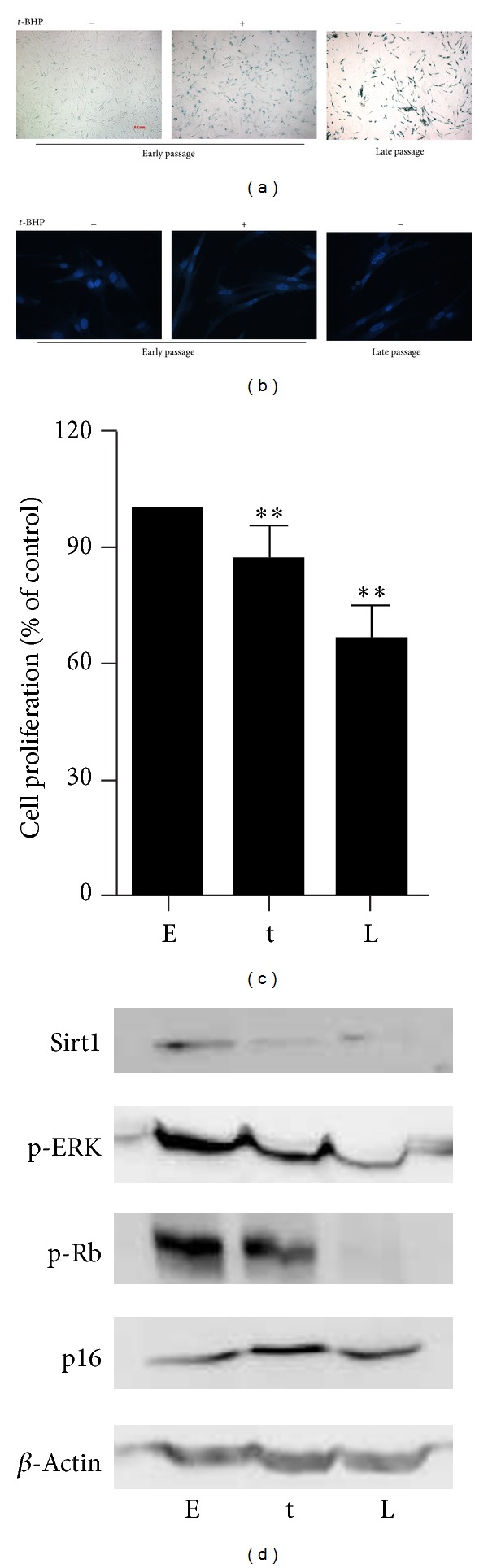
*t*-BHP treatment induced changes of senescence-associated features in WI38 cells. WI38 cells at early passage were treated by *t*-BHP to induce premature senescence; then SA *β*-gal staining (a), DAPI staining (b), cell proliferation assay (c), or Western blot assay using specific antibodies (d) were performed in early passage cells (E), *t*-BHP-induced premature senescent cells (t), and replicative senescent cells (L), respectively. Data were presented as mean ± SD of three independent experiments. **P < 0.01 compared with control group.

It was well accepted that long-term treatment with sublethal oxidative stresses can induce cells to senescent-like growth arrest, and it therefore has been frequently used for investigating cellular alteration due to oxidative stress-induced premature senescence [20–23]. In the current study, we examined premature senescence of WI38 HDFs after exposure to subcytotoxic dosage of *t*-BHP at 30 *μ*M for 5 d followed by 2 d recovery according to previous reports [21]. As shown in Figure 1, cells treated with *t*-BHP displayed features resembling replicative senescence as characterized by the enlarged cellular appearance, higher frequency of SA *β*-Gal staining, and appearance of SAHF in nuclei when compared to the early passage cells (Figures 1(a) and 1(b)). We also analyzed the effects of *t*-BHP on cell proliferation by MTT assay since a decrease in growth is another well-known feature of cellular senescence. We observed that *t*-BHP treatment decreased the proliferation of HDFs (Figure 1(c)). Furthermore, changes in various senescence-associated molecules were examined at the protein level.  Figure 1(d) showed that phosphorylation level of extracellular-signal-regulated kinases 1/2 (ERK1/2), which represents growth factor responsiveness, was decreased in both *t*-BHP-treated and senescent cells. The levels of p16 and p-Rb, the most well-known molecules in senescence HDFs, were also examined. Increased p16 expression, as well as decreased Rb phosphorylation, has been reported in senescent HDF cells [19]. Consistently, we observed changes in these proteins in the *t*-BHP-treated cells which were similar to senescent cells. All together, these data suggested that *t*-BHP exposure induced a replicative senescent-like phenotype in WI38 HDFs. 

### 3.2. BDMC Decreased Changes of Senescence-Associated Features in HDFs after *t*-BHP Treatment

**Figure 2:**
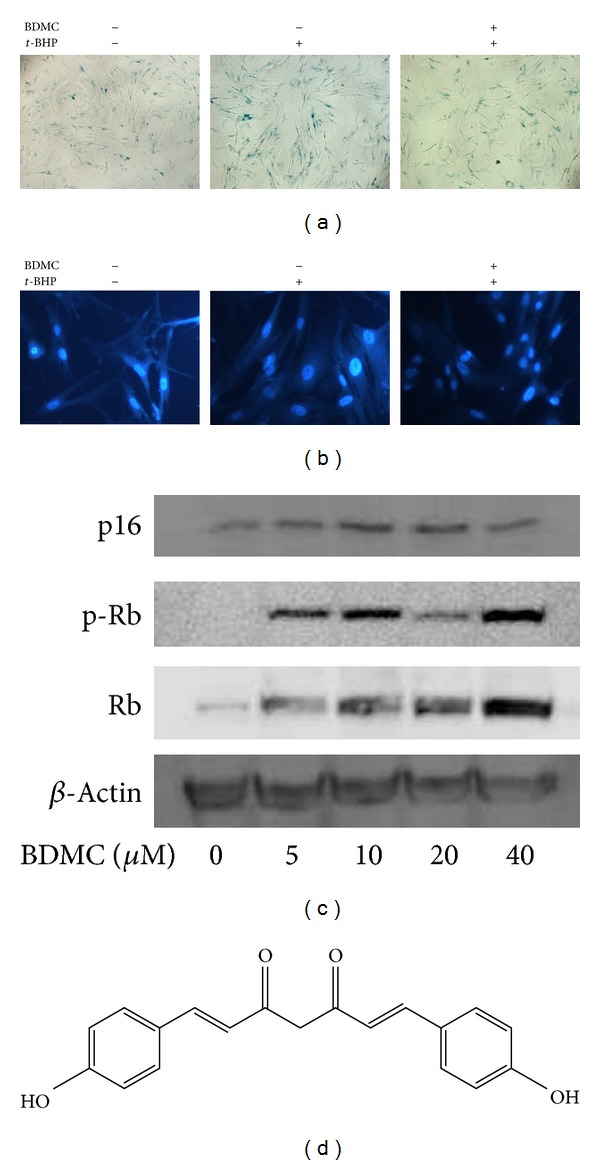
BDMC attenuated *t-*BHP-induced alterations of senescence-associated features in WI38 cells. WI38 cells at early passage were treated with 20 *μ*M BDMC for 48 h, followed by* t*-BHP exposure as described in Materials and Methods to induce premature senescence, and then SA *β*-gal staining (a) or DAPI staining (b) was performed. (c) Cells were treated with different dose of BDMC (5–40 *μ*M) for 48 h. Then, expressions of p16, p-Rb, and Rb were examined by Western blot assay. (d) The chemical structure of BDMC.

To investigate the prevention of BDMC on oxidative stress-induced premature senescence, HDFs were exposed to 20 *μ*M BDMC for 48 h before stimulation by *t*-BHP. As shown in Figure 2, *t*-BHP-treated WI38 HDFs showed an enlarged and flattened morphology, increased SA *β*-Gal positive staining, and SAHF accumulation which were consistent with earlier results. BDMC treatment was shown to markedly protect cells against *t*-BHP-induced senescence and shown only sporadic SA *β*-Gal positive staining and appearance of SAHF within the nuclei of WI38 cells as well. Furthermore, since the p16/Rb signaling pathway was indicated to play a crucial role in cellular senescence [24], we examined the effects of BDMC on p16 and its effector, retinoblastoma protein (Rb). HDFs were exposed to different concentrations of BDMC (5–40 *μ*M), and their effects on the expression and activation of p16 and Rb were analyzed. It was found that exposure of BDMC decreased p16 protein expression and increased the phosphorylation and total expression of Rb in a dose-dependent manner (Figure 2(c)). These results implied that p16/Rb pathway might be involved in the effect of BDMC in WI38 HDFs. 

### 3.3. BDMC Upregulated Sirt1 Expression in WI38 HDFs

**Figure 3:**
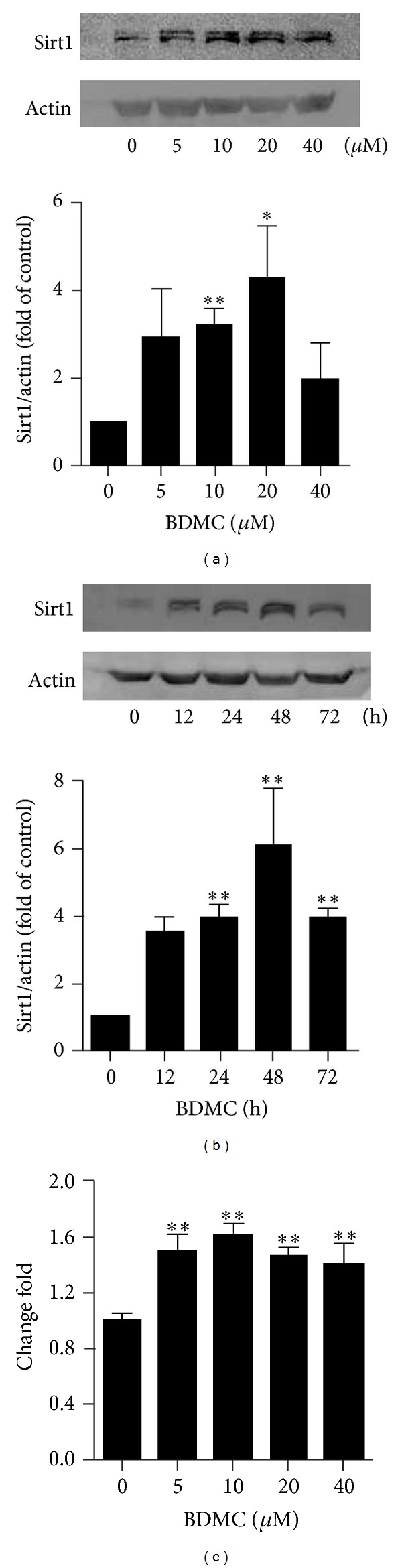
BDMC increased Sirt1 in transcriptional and translational levels in WI38 cells. (a) Dose response: cells were treated with different doses of BDMC (5–40 *μ*M) for 48 h. (b) Time response: cells were exposed to 20 *μ*M of BDMC for different duration (12–72 h). Then Sirt1 expression was measured by Western blot assay. (c) Cells were treated with different doses of BDMC (5–40 *μ*M) for 24 h; then total RNA was extracted, and reversed transcription, and real-time PCR were performed to assess Sirt1 gene level. Data were presented as mean ± SD of three independent experiments. *P < 0.05; **P < 0.01 compared with control group.

Sirt1 is well characterized for its beneficial effects against ageing-related diseases [19, 25–27]. Therefore, we examined whether BDMC could affect Sirt1 in WI38 HDFs. As shown in Figure 3(a), treatment with BDMC (5–40 *μ*M) for 48 h increased Sirt1 expression. A 12 h treatment with 20 *μ*M BDMC upregulated Sirt1 expression, while its expression was highest at 48 h posttreatment compared to control cells (Figure 3(b)). The upregulation of Sirt1 was also confirmed by real-time PCR analysis. The results indicated that Sirt1 mRNA in WI38 HDFs was elevated by BDMC as low as 5 *μ*M and displayed an approximately 1.6-fold increase at concentrations of 10 to 40 *μ*M (Figure 3(c)). These results indicated that BDMC may exert its effects by modulating Sirt1 expression in WI38 HDFs.

### 3.4. BDMC Activated AMPK in WI38 HDFs

**Figure 4:**
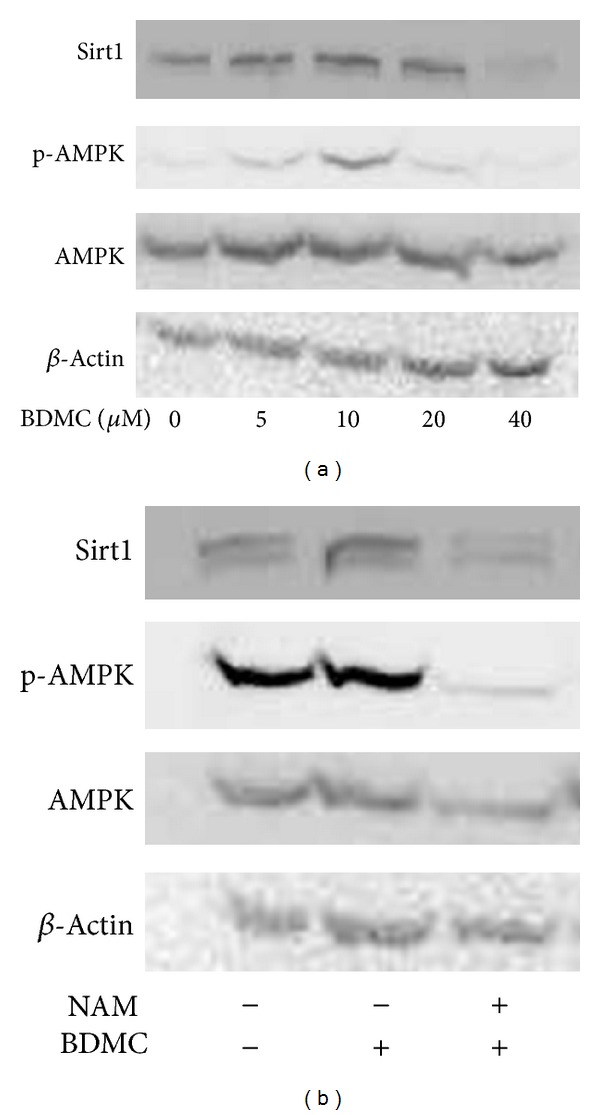
BDMC activated AMPK with the increase in Sirt1. (a) WI38 cells were treated with different doses of BDMC (5–40 *μ*M) for 48 h. Then, phosphorylation level of AMPK and total AMPK protein level were measured by Western blot assay. (b) WI38 cells were treated with nicotinamide (NAM) for 1-2 h, followed by BDMC (20 *μ*M) for 48 h; then Sirt1, p-AMPK, and AMPK were examined by Western blot assay.

AMP-activated protein kinase (AMPK), a crucial sensor for alterations in nutrients and intracellular energy levels, can be activated by caloric restriction, which is well accepted to improve age-related health and retard the ageing process [25, 28, 29]. In addition, it has been reported that Sirt1 is a key upstream activator of AMPK signaling [15]. After knowing that Sirt1 was affected by BDMC, we next examined whether BDMC could stimulate AMPK activation. HDFs were treated with different concentrations of BDMC (5–40 *μ*M) for 48 h, and then phosphorylation level of AMPK and total AMPK level were examined. As shown in Figure 4(a), BDMC increased the phosphorylation of AMPK without changing the total AMPK level, and the increase reached its maximal at 10 *μ*M. We next used a pharmacological inhibitor of Sirt1, nicotinamide (NAM), to confirm that activation of AMPK by BDMC was caused by the increase of Sirt1 in WI38 HDFs. We observed that NAM-treated HDFs at 40 mM [27], a concentration previously used to significantly decreased Sirt1 level, as well as block the activation of AMPK induced by BDMC (Figure 4(b)). These results suggested that AMPK stimulation by BDMC was mediated by Sirt1 increase. 

### 3.5. Protective Effects against *t-*BHP-Induced Senescence Are Partially Associated with the Alteration of Sirt1

**Figure 5:**
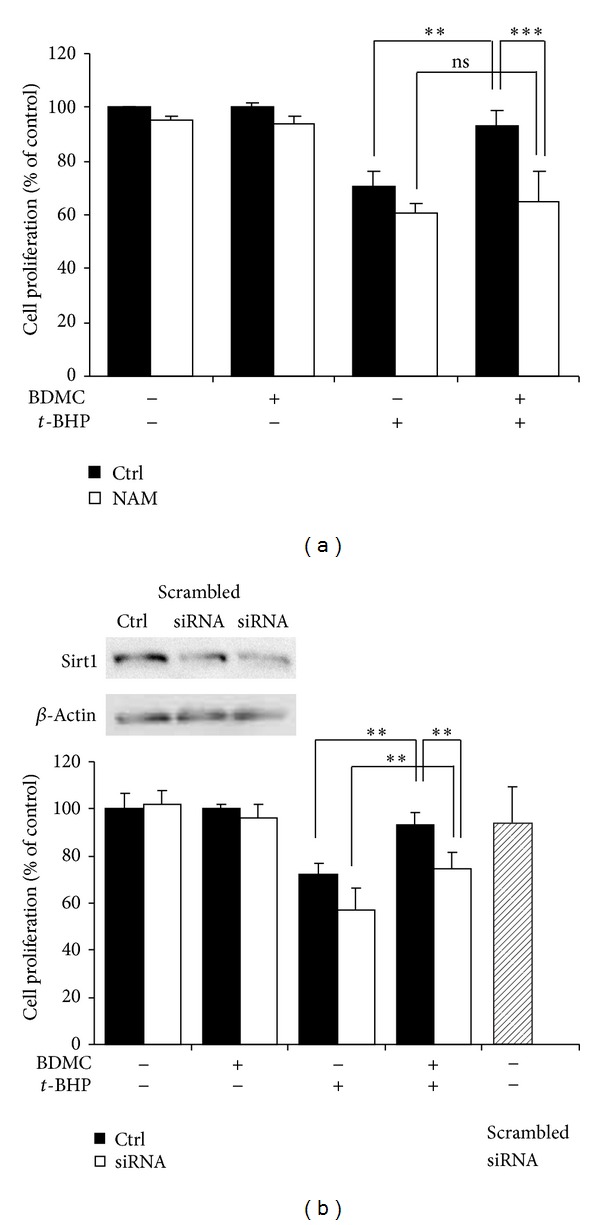
Inhibition in Sirt1 partially compromised the beneficial effects of BDMC on *t*-BHP-induced cellular senescence in WI38 cells. WI38 cells were pretreated with NAM (40 mM) for 1 h (a), or transfected with Sirt1 siRNA (b), then incubated in the presence or absence of BDMC (20 *μ*M) for 48 h, followed by exposure to* t*-BHP treatment. Cell proliferation was then determined by MTT assay. Data were presented as mean ± SD of three independent experiments. *P < 0.05; **P < 0.01.

To further explore the correlation between the protection of BDMC against *t*-BHP-induced premature senescence and the alteration in Sirt1 levels, we used the pharmacological inhibitor NAM or siRNA to decrease Sirt1 expression in WI38 HDFs. After transfection of Sirt1 siRNA or NAM treatment, WI38 HDFs were treated in the presence or absence of 20 *μ*M BDMC for 48 h, followed by stimulation by *t*-BHP. MTT assay was performed to assess cell proliferation. As shown in Figure 5, transfection with Sirt1 siRNA and pretreatment with NAM decreased Sirt1 protein levels with no cytotoxic effects observed when compared to the control. 

Cell proliferation was reduced to 77.7 ± 9.8% of the control after incubating with *t*-BHP. Cell proliferation was significantly reduced to 64.5 ± 7.8% and 57.3 ± 9.0% after treatment with *t*-BHP in siRNA-transfected or NAM-treated cells, respectively, compared to *t*-BHP-treated cells alone. This suggested that Sirt1 may protect against oxidative insults. Moreover, BDMC treatment attenuated *t*-BHP-induced loss of cell proliferation capability. However, this protection was partly compromised by the decrease of Sirt1 by either siRNA or NAM, suggesting that BDMC prevented the loss of proliferation via Sirt1 increase. However, other mechanisms may contribute to the effects of BDMC since BDMC still exerted slight protection in Sirt1 knockdown group. 

## 4. Discussion

Human diploid fibroblast cells can permanently lose their proliferative potential after a limited number of population doublings and enter replicative senescence [30]. Numerous lines of evidence have suggested that exposure to exotic oxidative stress can trigger stress-induced premature senescence in HDFs within a few days. This premature senescence resulted from accumulation of random damage and loss of repair function caused by reactive oxygen species (ROS), and shared common features with replicative senescence [31]. In this study, we analyzed the long-term effects of sublethal oxidative insults using *t*-BHP in WI38 HDFs. In line with previous reports, treatment with *t*-BHP induced senescence of HDFs, indicated by an enlarged cellular appearance, a higher frequency of SA *β*-Gal staining activity, accumulation of SAFH in nucleus, and changes in the molecular markers including p16 and Rb. 

Data obtained from laboratory experiments and clinical trials showed the potential of BDMC to improve age-associated disorders. BDMC has been reported to protect endothelial cells against *t*-BHP-induced cell damage [32], by inducing hemo oxygenase-1 expression through decreasing nitric oxide synthase expression and nitric oxide production [33]. In addition, study in patients with sporadic Alzheimer's disease showed that BDMC could improve the innate immune system and increase amyloid-beta clearance from the brain [34]. In this study, we examined the effects of BDMC on oxidative stress-induced senescence in WI38 HDFs. Our results indicated that pretreatment with 20 *μ*M BDMC could attenuate *t*-BHP-induced changes of HDFs, including decrease in SA *β*-Gal positive staining and appearance of SAHF. Most of the reports have attributed the protection of curcuminoids to their maintenance on cellular redox homeostasis. On one hand, curcuminoids are well known for their promising antioxidative effects due to the phenolic hydroxyl group in their molecular structure. On the other hand, it has been demonstrated that curcuminoids could induce the expression of antioxidant enzymes such as superoxide dismutase and heme oxygenase-1 and thereby exert cytoprotective effects in response to oxidative insults. It is interesting to find in our study that BDMC dose dependently regulated p16/Rb, one of the major senescence-related pathways, suggesting that ROS-irrelevant mechanisms may also contribute to the protective effects of BDMC against oxidative insults. 

Much interest has been shown of Sirt1 due to its essential role in metabolism, longevity, and stress response. It has been demonstrated that caloric restriction retards ageing process by modulating Sirt1 function in many species ranging from yeast to primates. An increase in Sirt1 protein by genetic methods or pharmacological activators can extend the lifespan of organisms and prevent cellular senescence [8, 16, 24, 35]. Our data in Figure 1(d) showed that, similar to the cells of late passage, Sirt1 level was also decreased in *t*-BHP-induced premature senescent cells as compared with cells of early passage, suggesting that this protein may take part in the oxidative stress-induced premature senescence. In addition, it was reported that some polyphenols conferred protection against oxidative stress-induced cell apoptosis and senescence through activation of Sirt1 [10, 36]. Therefore, we hypothesized that Sirt1 might also be involved in the protective effects of BDMC against *t*-BHP-induced premature senescence in HDFs. Our results demonstrated that both Sirt1 protein and mRNA level increased upon treatment with BDMC. Meanwhile, AMPK, which is a sensor of cellular energy status, was also up regulated by BDMC with the increase of Sirt1. This observation is consistent with previous reports that demonstrated curcuminoids increased AMPK phosphorylation and its downstream factor acetyl-CoA carboxylase in hepatoma cells [37]. Additionally, AMPK activation has been shown to regulate curcumin-stimulated glucose uptake in myotube cells [38]. These data raised the possibility that curcumin-activated AMPK is involved in cell metabolism and thereby may be related to BDMC-increased Sirt1. Recent studies in hepatocytes indicated that activation of Sirt1 stimulated the basal AMPK signaling pathways and prevented lipid accumulation [15, 39], suggesting that AMPK is an important downstream target of Sirt1. Additionally, AMPK activation was suggested to be a crucial mechanism for polyphenol-mediated effects [15, 40]. Therefore, we explored whether BDMC activated AMPK in a Sirt1-dependent manner. For this, a Sirt1 inhibitor NAM was used in the study. The results showed that treatment with BDMC led to an increase of p-AMPK after 48 h treatment. However, this increase could not be observed in the NAM pretreatment cells, indicating that BDMC-induced AMPK activation was dependent on Sirt1. 

We further explored whether the beneficial effect of BDMC against *t*-BHP-induced premature senescence was through the increase of Sirt1. Sirt1 protein in WI38 HDFs was inhibited by siRNA or NAM, and then these cells were incubated with BDMC followed by *t*-BHP-induced senescence. Consistent with previous reports, our results demonstrated that cells with decreased level of Sirt1 were more sensitive to* t*-BHP-induced damage than normal cells, indicating that Sirt1 plays a role in protecting against oxidative insults and decreased growth. Moreover, we found that the protective effects of BDMC were partly blocked in cells with decreased level of Sirt1. This observation provided strong support that BDMC exerted its preventive effects on *t*-BHP-induced senescence-like growth arrest by increasing Sirt1. However, although this protective effect was attenuated after downregulation in Sirt1, BDMC still exhibited partial protection to WI38 cells. This may be due to residual Sirt1 level that was not completely abolished by the pharmacological inhibitor or siRNA. Furthermore, this suggests that other mechanisms may be involved, which contribute to the protective effects of BDMC. Further studies are needed to identify additional mechanisms, such as the enhancement of antioxidant defenses. 

Overall, our study indicated that BDMC prevented *t*-BHP-induced senescence-like growth arrest in WI38 HDFs. We further showed that this might be dependent on the increase of Sirt1 and subsequent activation of AMPK. This is to our knowledge the first report linking Sirt1 with the protective effects of BDMC against oxidative-stress-induced cellular senescence in WI38 fibroblast cells. Although other mechanisms, such as antioxidative pathways, could not be excluded, the link between BDMC and Sirt1 provides novel insight into the therapeutic potential of BDMC as well as curcuminoids. Apart from the important role in longevity, Sirt1/AMPK has also been regarded as a crucial link between cell metabolism and stress response. Thus, this study also provides possibility that regulation on Sirt1 could be the underlying mechanisms of other beneficial effects of curcuminoids. 

## Abbreviations

BDMC: Bisdemethoxycurcumin
HDF: Human diploid fibroblast
SA-*β*-Gal: Senescence-associated *β*-Gal
SAHF: Senescence-associated heterochromatic foci
ROS: Reactive oxygen species
BME: Basal medium Eagle medium
FCS: Fetal calf serum
DAPI: 4′-6-Diamidino-2-phenylindole
MTT: 3-(4,5-Dimethyl-2-thiazolyl)-2,5-diphenyl tetrazolium bromide
Rb: Retinoblastoma protein
ERK: Extracellular-regulated protein kinases.
